# The complete mitochondrial genome of *Amphinemura bulla* Shimizu, 1997 (Plecoptera: Nemouridae) from Japan

**DOI:** 10.1080/23802359.2021.1884029

**Published:** 2021-03-15

**Authors:** Ying Wang, Jinjun Cao, Dávid Murányi, Xiling Chen, Fenming Yan

**Affiliations:** a College of Plant Protection, Henan Agricultural University, Zhengzhou, China; b Postdoctoral Research Base, Henan Institute of Science and Technology, Xinxiang, China; c Plant Protection Institute, Centre for Agricultural Research, Hungarian Academy of Sciences, Budapest, Hungary; d Department of Zoology, Hungarian Natural History Museum, Budapest, Hungary

**Keywords:** Mitochondrial genome, phylogenetics, *Amphinemura bulla*

## Abstract

The genus *Amphinemura* belongs to the family Nemouridae (Plecoptera) and has 205 species in the Holarctic and Oriental Regions. We sequenced the fourth complete mitochondrial genome of *A. bulla* Shimizu, 1997. The mitogenome is 15,827 bp long with 37 genes plus a control region with an A + T content of 68.9%. There are 10 intergenic spacers (75 bp total) and 13 gene overlaps (43 bp total). All protein-coding genes (PCGs) use normal initiation codons, except *ND1* and *ND5* which begin with TTG and GTG. Two PCGs (*COII* and *ND5*) use a single T as a partial termination codon. Phylogenetic analyses showed that *Nemoura* and *Amphinemura* were sister group resulting in a paraphyletic Amphinemurinae different from the morphological classification.

Baumann (1975) divided the plecopteran family Nemouridae into two subfamilies, the Nemourinae and the Amphinemurinae. To date, there are 205 valid species in the subfamily Amphinemurinae from the Holarctic and Oriental Regions with more than 90 species distributed in China (Yang and Li 2018; DeWalt and Ower 2019; DeWalt et al. 2020; Li et al. 2020; Mo et al. 2020). Three mitochondrial genomes sequenced from the genus *Amphinemura* are all from Chinese species (Cao et al. 2019; Chen et al. 2020), here, we sequence a fourth complete mitochondrial genome, *Amphinemura bulla* Shimizu, 1997 (GenBank accession number MW339348) from Japan using high-throughput sequencing. An adult specimen was collected from Matsuyama City, Ehime Prefecture, Japan (coordinate as follows: N°33.516, E°133.500) on 22 October 2015 by Dávid Murányi. The voucher specimen (no. VHL-0002) was deposited in 100% ethanol and stored in the Entomological Museum of the Henan institute of Science and Technology (Wang Ying, wangying198586@163.com), China. Total genomic DNA was extracted from muscle using the QIAamp DNA Blood Mini Kit (QIAGEN, Hilden, Germany). Illumina Hiseq 2500 with 500 cycles of paired-end sequencing (250 bp reads) was performed at BerryGenomics Co., Ltd. (Beijing, China). Sequence reads were assembled using the BioEdit version 7.0.5.3 (Hall 1999) and annotated using the MITOS Web Server (Bernt et al. 2013).

The complete mitochondrial genome of *A. bulla* is a circular double-stranded with 15,827 bp in length, which encodes 22 transfer RNA genes (tRNAs), 13 protein-coding genes (PCGs), two ribosomal RNA genes (rRNAs), and a control region, were similar with other published stoneflies (Cao et al. 2019; Ding et al. 2019; Chen et al. 2020). The base composition of the mitochondrial genome was biased toward A and T, with 68.9% A + T content (A = 36.5%, T = 32.5%, C = 18.9%, G = 12.2%). The A + T content of PCGs, tRNAs, rRNAs, and the control region were 67.2, 70.6, 71.0%, and 82.0%, respectively. There were 10 intergenic spacers which ranged from 1 to 26 bp in size (75 bp total) and 15 gene overlapping gene regions which range from 1 bp to 8 bp (43 nucleotides). All but two of PCGs in *A. bulla* mitochondrial genome used standard invertebrate start codons ATN while *ND1* and *ND5* used TTG and GTG. Eleven PCGs used the canonical termination codons TAA and TAG, while two PCGs (*COII* and *ND5*) used incomplete termination codons (T).

Phylogenetic reconstruction was performed based on the concatenated 13 PCGs from 19 published plecopteran species plus *Amphinemura bulla* (Figure 1). *Mesocapnia arizonensis* and *Mesocapnia daxingana* served as outgroups. The Bayesian inference (BI) and maximum-likelihood (ML) methods obtain the same topology tree. The phylogenetic relationship between the subfamilies Nemourinae and Amphinemurinae is controversial. *Nemoura* and *Amphinemura* were sister groups which is not consistent with the correct morphological classification (Zwick 2000). It is worth adding more species to improve the phylogeny of the stoneflies.

**Figure 1.**
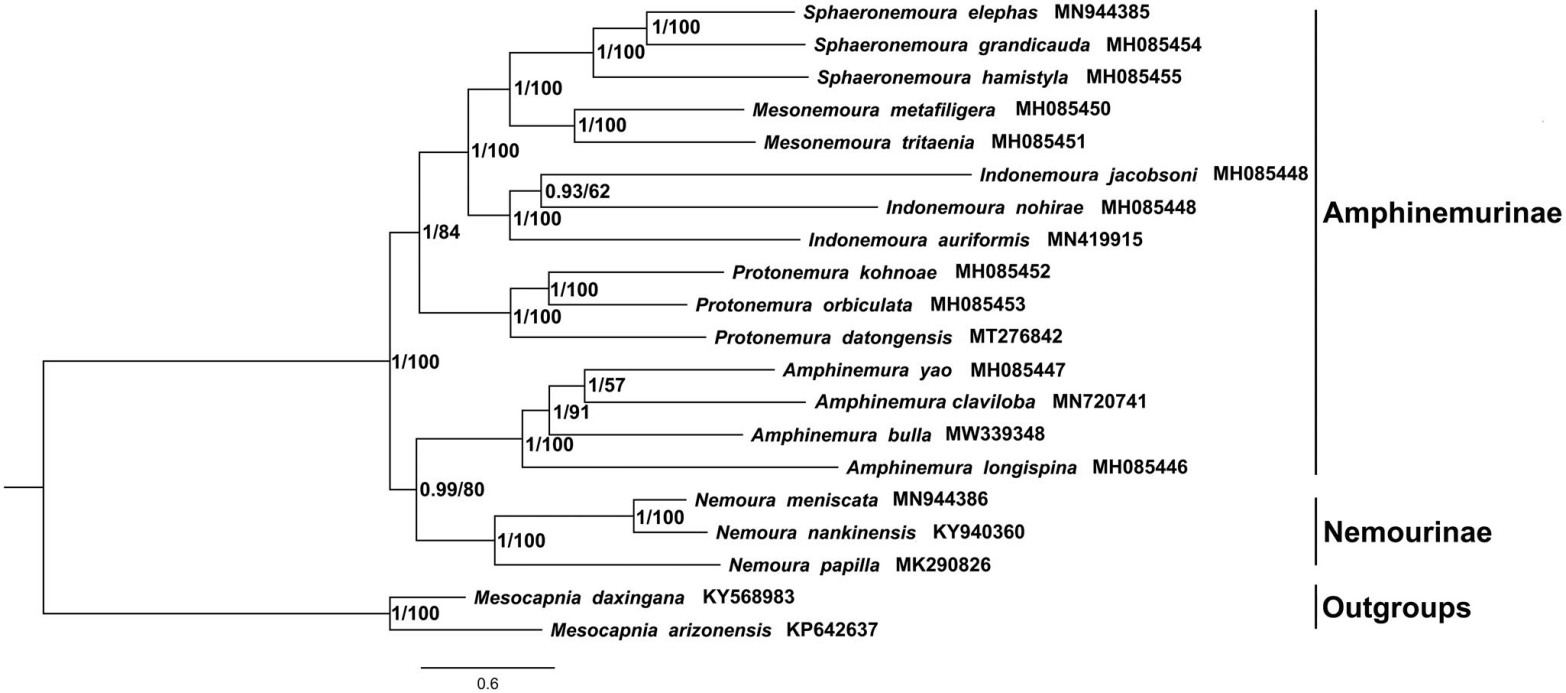
The phylogenetic trees based on the concatenated nucleotide sequences of 13 PCGs by the Bayesian inference (BI) and maximum-likelihood (ML) methods. The complete mitochondrial genomes of *Amphinemura bulla* and 19 other stoneflies are available from GenBank. Scientific name is followed by the accession number for each species.

## Data Availability

The genome sequence data that support the findings of this study are openly available in GenBank of NCBI at https://www.ncbi.nlm.nih.gov/ under the accession no. MW339348.
